# Evaluation of a High Throughput Starch Analysis Optimised for Wood

**DOI:** 10.1371/journal.pone.0086645

**Published:** 2014-02-11

**Authors:** Chandra Bellasio, Alessio Fini, Francesco Ferrini

**Affiliations:** 1 Department of Animal and Plant Sciences, University of Sheffield, Sheffield, United Kingdom; 2 Department of Plant Sciences, University of Cambridge, Cambridge, United Kingdom; 3 Department of Agri-Food Production and Environmental Sciences – section Woody Plants, University of Florence, Sesto Fiorentino (Florence), Italy; Iowa State University, United States of America

## Abstract

Starch is the most important long-term reserve in trees, and the analysis of starch is therefore useful source of physiological information. Currently published protocols for wood starch analysis impose several limitations, such as long procedures and a neutralization step. The high-throughput standard protocols for starch analysis in food and feed represent a valuable alternative. However, they have not been optimised or tested with woody samples. These have particular chemical and structural characteristics, including the presence of interfering secondary metabolites, low reactivity of starch, and low starch content. In this study, a standard method for starch analysis used for food and feed (AOAC standard method 996.11) was optimised to improve precision and accuracy for the analysis of starch in wood. Key modifications were introduced in the digestion conditions and in the glucose assay. The optimised protocol was then evaluated through 430 starch analyses of standards at known starch content, matrix polysaccharides, and wood collected from three organs (roots, twigs, mature wood) of four species (coniferous and flowering plants). The optimised protocol proved to be remarkably precise and accurate (3%), suitable for a high throughput routine analysis (35 samples a day) of specimens with a starch content between 40 mg and 21 µg. Samples may include lignified organs of coniferous and flowering plants and non-lignified organs, such as leaves, fruits and rhizomes.

## Introduction

Trees store non-structural carbohydrates (NSC) in wood parenchyma with starch constituting the main NSC for long-term reserves [1], [2]. Starch is mobilized to maintain basal metabolism during winter, support vegetative growth in spring, and to cope with energy deficits during the growing season [3], [4], [5], [6]. Starch content (SC) has been related to ecological performance [7], [8], [9], and physiological [10], [11] and productive traits [12], [13], and is therefore an important indicator for studying plant responses. Although starch is the most important long-term carbohydrate reserve, starch dynamics and physiology of reserves are still confined to specialized studies.

SC is normally quantified by a destructive analysis whereby the sample is ground and starch is hydrolysed to glucose. Hydrolysis of starch has a critical importance in the analysis (an introductory overview of starch analysis is reported in Box 1). Acid hydrolysis is not suitable for wood matrix because the process cleaves structural carbohydrates into monomeric sugars that result in high interference [14], [15], [16], [17], [18]. Enzymatic hydrolysis can target specific NSC and it has been widely used [17], [19], [20], [21], [22], [23], [24], [25], [26], [27], but only a few studies have addressed SC of wood [20], [21], [26], [28]. These are subject to technical limitations that include slow purification procedures [22], long digestion times [20], [21], [29], a neutralization step [23], the requirement for separate gelatinisation with KOH [26]. Others were not aimed at detailing the instructions for SC analysis [15], [24], [25]. Furthermore, in some cases, the enzymes cited are no longer available from the vendor's catalogue, e.g. [26]. As a consequence, it is not clear what is the most efficient and appropriate protocol for starch analysis in wood. Due to practical constraints, there is a trade-off between analytical throughput and performance.

This study aims to identify, optimise, and characterise the performance of a protocol for starch analysis in wood and to present it in detail for easy replication. The performance of an analysis is measured in terms of accuracy and precision. Accuracy is the closeness of agreement between a measured quantity and the true value of that quantity [30]. For instance, accuracy is important when a trend is being studied (e.g. time courses), when results from different groups are compared, or when other quantities are derived from the measured SC. In this paper, we express accuracy as systematic error (also known as trueness or bias), i.e. the deviation between the detected SC and the actual value of SC. An important effect on accuracy is brought about by interferents, which exert a systematic effect on the detection of starch. This ‘interference’ is computed as the difference between SC detected in a reference analysis and in an analysis where the interferent is present. Precision is the closeness of agreement between measured values obtained by replicate measurements [30]. Precision is important in hypothesis testing (e.g. treatment vs. control), whereby precision correlates with the power of the statistical test. In this paper we numerically express precision with the measurement ‘dispersion’ (i.e. variance, standard deviation, S.D., and coefficient of variation C.V.), hence high precision is represented by low values for dispersion. Factors influencing precision directly affect variance. A key factor in analytical procedures is the day effect, which is the fraction of the total variance ascribable to daily fluctuations. Unlike other components of variance, when the experiment is purposely designed, the day effect can be isolated. The isolation of the day effect decreases the residual error and increases the precision of the analysis.

Commercial kits for routine SC analysis used for food and feed [18] represent a suitable candidate for optimisation. These analytical procedures are cheap, fast and repeatable [27]. Kits contain pure and certified enzymes with engineered pH optima (and hence do not require pH adjustment). The availability of thermostable amylase allows the coupling of starch gelatinization and hydrolysis. For these reasons, such protocols are high throughput and straightforward, they have been validated by inter-laboratory studies [31], and often support is made available by the vendor. However, these protocols are not optimised for the analysis of wood. The analysis of SC in wood is more problematic than the equivalent analysis in food. Issues associated with the analysis of wood include: i) starch exists in smaller concentrations that are locked within a matrix of structural polysaccharides (SC in food often exceeds 50%, whereas SC in wood can be below 1%), ii) lower reactivity of wood starch, and iii) large amounts of extractable compounds in wood (resins, gums, oils, terpenes [32], [33]). These characteristics require dedicated experimental design and data analysis.

By optimizing a standard method (AOAC 996.11 and 76.13 [34]) we achieved 3% accuracy and precision. The interfering effect of matrix polysaccharides and wood type (three different organs of four different species) was generally negligible. The analytical performance of the optimised protocol is suitable for high throughput routine analysis of starch in lignified (e.g. roots, twigs, mature wood) as well as non-lignified (e.g. fruits, leaves, rhizomes) samples of coniferous and flowering plants.

**Box 1. Brief overview of starch analysis**

The available methods for starch analysis can be described as consisting of five steps:

**Separation and removal of soluble carbohydrates from the wood matrix.** The solvent used can be water, an aqueous solution of ethanol, or a mixture of methanol, chloroform, and water.

**Gelatinization of starch granules.** To allow a fast subsequent cleavage, granules are swollen and solubilized, yielding water-soluble swollen granules. However, granules are not hydrolysed in this step. Generally, gelatinization involves treatment with hot water or with diluted KOH. In several cases, gelatinization has been coupled with the previous step by treating with hot ethanol. Starch granules are not soluble in hot ethanol, and sugars can therefore be washed together with the supernatant while swollen starch granules remain in the sediment and can be solubilized upon subsequent water addition.

**Starch selection from interfering carbohydrates.** Some acid methods entail starch isolation by precipitation with iodine. Not all acid methods have a selective step, and thus they often result in low selectivity because both structural carbohydrates and starch are hydrolysed by concentrated acids. Enzyme methods rely on the inherent specificity of the catalyst [18], which is superior to the precipitation selectivity [16].

**Hydrolysis of starch to glucose.** Starch can be hydrolysed by either using an acid solution (35% perchloric acid or 1 M HCl or 0.1 M H_2_SO_4_, according to different protocols, [16] [14] [15]) or using a combination of enzymes. In the latter case, the quantitative conversion of starch or dextrins to glucose is always performed by amyloglucosidase. This treatment could be the only hydrolysis [21], [24] or it can be preceded by a pre-treatment with α-amylase to break starch into oligosaccharides and dextrins [35] to speed up the amyloglucosidase digestion. The availability of thermostable amylase has allowed the coupling of gelatinization with amylase treatment in boiling acetate buffer (100°C) [36]. Enzymes with engineered pH optima do not require different buffers for the two reactions (e.g. Box 2). Since enzymatic methods do not require neutralization, they can be followed directly by an enzymatic glucose analysis.

**Glucose analysis.** Glucose can be assayed by means of a direct reaction between glucose and a dye as in the copper iodometric technique [37] and the anthrone method [26], [38], [39], [40]. These methods use concentrated reagents so they do not suffer from the residual mineral acid in the sample. For this reason they are preferred when following an acid hydrolysis. Alternatively, in enzymatic methods the reaction between glucose and a dye is enzyme-mediated. The most common enzymatic glucose assay involves a peroxidase-catalysed reaction where glucose is oxidized to gluconic acid with quantitative production of hydrogen peroxide. H_2_O_2_ in turn oxidizes a dye (ortho-dianisidine or 4-amynoantipyrine) in a quantitative enzyme-catalysed reaction. Enzymatic methods are preferred when analysing samples containing interferents (as with wood samples, containing a wide array of matrix polysaccharides) because of the high selectivity of the enzymes [26]. Some authors report chromatographic methods e.g. [19], which generally require long setup and sample purification.

## Materials and Methods

### Starch analysis

**Figure 1:**
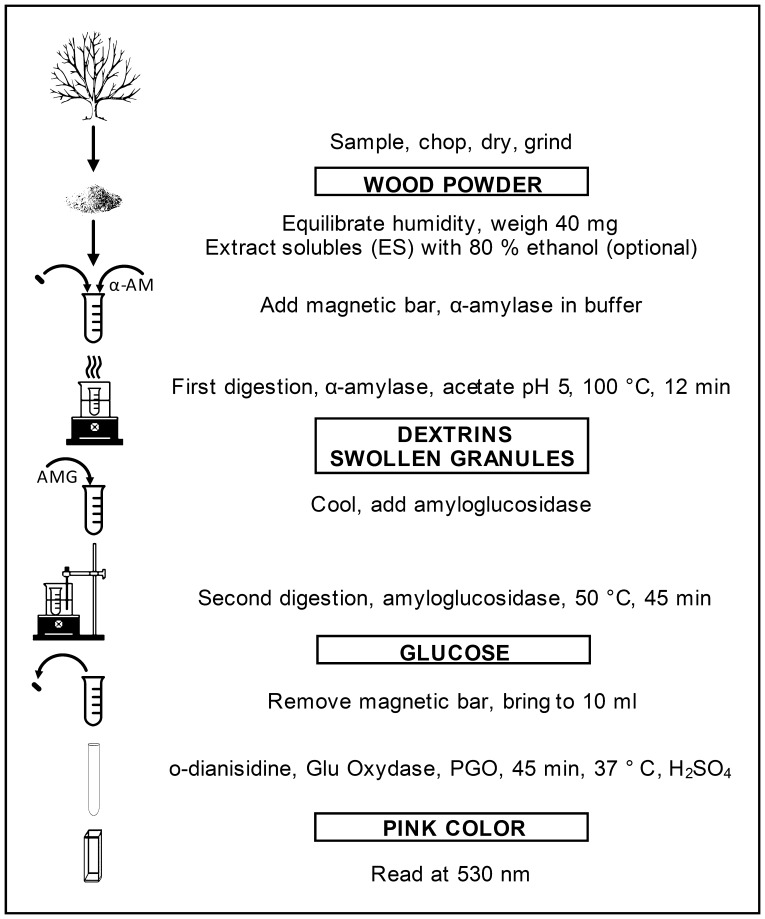
Schematic of starch analysis.

Starch was quantitatively determined by a destructive analysis consisting of wood grinding (with optional extraction of solubles, ES), a two-step digestion of starch, and glucose assay (Figure 1). The digestion has the pivotal role of *quantitatively* converting starch into glucose, hence it was carefully optimised. The optimisation phase (240 starch determinations) is not shown in the Results, but briefly described here. Reaction conditions (temperature, duration, and enzyme concentrations) of the standard method AOAC 996.11 and 76.13 [18], [34] were modified until the analytical performances on standard corn starch and wood samples were similar. In the optimisation we found that the addition of a magnetic bar in each tube [14], [26] resulted in 28% higher starch detection. This was most likely a consequence of facilitating the action of hydrolysing enzymes on starch granules on the suspended slush. For this reason the magnetic bar was always added in the optimised digestion.

The complete optimised protocol is reported in detail in Box 2 and the required reagents and solutions are listed in Box 3. In summary, dry wood powder was digested during the first step at 100°C (Figure 1) in which starch granules were swollen and partially cleaved by thermostable α-amylase, and in the second step, amyloglucosydase quantitatively converted the products of the first reaction into glucose. After the digestion, glucose was analysed with an enzymatic assay optimised for wood samples [26], [41]. In this assay, glucose was enzymatically oxidised to gluconic acid, and the hydrogen peroxide produced, in turn, enzymatically oxidised ortho-dianisidine in a stoichiometric relationship. Sulphuric acid was added to stop the reaction, stabilize the pink colour, and shift the absorption peak away from plant pigment interference.

**Box 2. Optimised starch analysis**

**Sample preparation.** Chop wood samples in pieces and dry in oven (e.g. 70°C for 2 days). Grind dry wood to yield impalpable wood powder without evident fibre structure. In this study approx. 1.3 g of dried wood pieces were ground in a 10 ml stainless steel jar and bead set (Qiagen, NL, product number 69985) with a mixer mill (tissue lyser Retsch, D) at 25 Hz for 210 s. Keep the wood powder in oven at 70°C for at least 12 h, cool in desiccator for 30 min and immediately weigh approx. 40 mg of sample in screw capped round bottom centrifuge tubes (e.g. TPP, Trasadingen, CH, product 91016) to an accuracy of ±0.1 mg. Include sample blank, SAB, a tube with no powder. Moisten the powder with 0.2 ml of 80% ethanol or extract solubles three times with 5 ml of 80% ethanol (optional, see Discussion) by stirring for 5 min and centrifuging (9000 g for 5 min). The supernatant may be discarded or collected for sugar analysis (e.g. [21], [24], [42]).

**Digestion.** Add to each tube containing the ethanol-moistened wood powder 3 ml of α-amylase in buffer (Solution 1, Box 3) and a magnetic bar (8 mm×3 mm) to suspend slush during digestion. Vortex and incubate for 12 min in boiling water on a magnetic stirrer (e.g. RCT Basic IKA, Staufen, D). Ensure tubes are submerged and bars spinning throughout. Cool the tubes in tap water for 2 min. Add 0.15 ml of amyloglucosidase (Bottle 2, Box 3). Vortex to break lumps and incubate at 50°C for 45 min in water bath (avoid dry incubators) on a magnetic stirrer (e.g. as above, with thermostat). Remove bar, adjust volume to 10 ml adding water to reach mass of (tube with dry wood powder) + 10.0 g. Mix thoroughly and centrifuge at 9000 g for 5 min at room temperature. Use the supernatant for glucose assay.

**Glucose assay.** Label 5 ml test tubes for samples (S), sample blank (SAB), glucose standards (GS, in triplicate) and standard blank (STB). Add water: 540 µl to S and SAB, 580 µl to GS, and 600 µl to STB. Add 60 µl of sample supernatant to S, 60 µl of sample blank supernatant to SAB, and 20 µl of glucose standard solution to GS. Add 2 ml of Reagent 3 to all tubes (Table 1). Mix thoroughly avoiding foam formation and incubate in water bath at 37°C for 45 min (avoid dry incubators). Stop the reaction by adding 400 µl of H_2_SO_4_ 75% in the same order followed to start the reaction with Reagent 3. Mix thoroughly and read absorbance at 530 nm. When the assay is out of linearity the addition of H_2_SO_4_ forms a cloudy brown suspension, in that case, a part of the supernatant in S and SAB can be replaced with water.

**Table 1:** Dilution table for the glucose determination.

	Water	Supernatant	Reagent 3	Standard	H_2_SO_4_ 75%	Total Volume
Sample (S)	540	60	2000	-	400	3000
Sample Blank (SAB)	540	60	2000	-	400	3000
Glucose Standard (GS)	580	-	2000	20	400	3000
Standard Blank (STB)	600	-	2000	-	400	3000

Volume is expressed in µl.


**Calculation.** SC was calculated as:
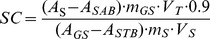
(1)

Where: SC is expressed in µg/mg; the various *A* are the absorbances of the specimens listed in Table 1 (for subscripts refer to Table 1); *m_GS_* is the mass of glucose added to GS (20 µg); *V_T_* is the total digestion volume to which tubes were adjusted (10000 µl); *m_S_* is the mass of wood powder weighed, in mg; *V_S_* is the supernatant volume used in the glucose assay, (60 µl); 0.9 converts mass of glucose to mass of starch.

A step-by-step example of analysis design and data treatment is reported in Supporting Information.

**Box 3. Reagents and solutions for starch digestion and glucose assay**

**Bottle 1.** Pure thermostable α-amylase in stabilized solution (E-BLAAM, Megazyme international, IR), with assayed specific activity (on p-dinitrophenyl-α-D-maltoheptaoside at pH 6 and 40°C) of 54 U mg^−1^ protein and concentration of 3000 U ml^−1^. Stable for 4 years at 4°C.

**Bottle 2.** Pure amyloglucosidase from Aspergillus niger in 50% glycerol and 0.02% sodium azide (E-AMGDF, Megazyme international, IR), with assayed specific activity (on soluble starch at pH 4.5 and 40°C) of 3260 U ml^−1^. Stable for 4 years at 4°C

**Buffer.** Sodium acetate buffer 0.1 M pH 5.0 plus CaCl_2_ 5 mM. Add 5.8 ml of glacial acetic acid (1.05 g ml^−1^) to 900 ml of deionized water. Adjust the pH to 5.0 with 1 M NaOH (4 g in 100 ml), approx. 50 ml is required. Add 0.74 g of CaCl_2_ dihydrate and dissolve. Adjust the volume to 1 L. Stable for 2 months at 4°C.

**Solution 1.** α-amylase in acetate buffer. Dilute 1.0 ml of the content of Bottle 1 + 29 ml Buffer. The solution can be stored at 4°C for up to a week. Stable for up to 3 years at −20°C.

**H2SO4 75%.** Slowly add 75 ml of fuming sulphuric acid + 25 ml of deionized water in a heavy glass bottle preferably in an ice-water bath to speed cooling.

**o-dianisidine solution.** Dissolve 50 mg of purified o-dianisidine for use with peroxidase and peroxidase-coupled reactions (Sigma product number D3252) in 20 ml deionized water. Note: o-dianisidine is carcinogenic, handle the powder and the solution with appropriate safety equipment.

**Reagent 3.** Complete reagent for glucose assay. Contains of 500 U of glucose oxidase (Aspergillus niger) and 100 purpogallin units of peroxidase (horseradish) in 0.1 M phosphate buffer pH 7.0 plus o-dianisidine. Dissolve the content of a capsule of PGO reagent kit (Sigma product number P7119). Open the capsule in a dark bottle, discarding the gelatine sheath, add 100 ml of deionized water and allow complete dissolution. Add 1.6 ml of o-dianisidine solution.

**Standard.** Glucose solution 1 µg µl^−1^ in benzoic acid 0.1%. The standard can be either purchased (Sigma, US, product G6918) or prepared. Dry standard grade glucose in oven at 80°C until constant weight is reached. Cool to room temperature in a desiccator. Weigh precisely 250.0 mg of glucose and transfer quantitatively in a 250 ml volumetric flask. Add 250 mg of benzoic acid and water (120 ml are needed to dissolve the benzoic acid); wait for dissolution. Bring to 250 ml with deionized water. Mix thoroughly. Stable for 6 months at 4°C.

### Evaluation of starch analysis and glucose assay performance

#### Accuracy

Accuracy was evaluated by analysing samples of known SC (SKSC). To prepare SKSC, pure cellulose (Sigma, US, reagent number 8002) and pure corn starch (standard grade, Sigma, US, product number S5296) were dried at 70°C and cooled in a desiccator. 10 g/100 g SKSC was prepared by weighing accurately 1800.0 mg of cellulose and 200.0 mg of starch and homogenizing the mixture at 25 Hz for 90 s in a mixer mill. (1.25, 2.5 and 5) g/100 g SKSC were prepared by subsequent dilution of the former with pure cellulose, homogenizing as described. Starch was analysed seven times and accuracy was computed as systematic error in absolute and relative terms.

Accuracy was also evaluated with the indirect comparative approach used by Rose [16]. The optimised analysis was compared to an enzymatic analysis (Sigma STA20 [41]). We have chosen STA20 because information and reagents are available worldwide, and it uses purified and certified enzymes and therefore does not suffer matrix interference (the alternative acid-based procedures are not suitable for wood matrix analysis [14], [15], [16], [17], [43]). Furthermore, we were very familiar with STA20 as we extensively used it as reference in the optimisation phase. STA20 had stable performance on pure starch, comparable to the standard method. In STA20, 50 mg of wood powder was treated for 5 min with purified thermostable α-amylase (Sigma product A4582) at 100°C. Tubes were cooled and adjusted to 10 ml. 1 ml of such digestate was incubated with purified amyloglucosidase (Sigma product S9144) for 15 min at 60°C and used for the glucose assay as described above. For the comparison, 28 samples of *Acer pseudoplatanus* L. twigs were collected, chopped, dried (70°C, 72 h), and ground, as described in sample preparation (Box 2). Starch was analysed in quadruplicate (optimised) and in quintuplicate (STA20) for a total of 252 determinations.

#### Interferents

The interference of **matrix polysaccharides** was evaluated by analysing seven times the SC of pure cellulose (described above) and pure pectin (Sigma, US, reagent number P8471), which are the two constituents of wood matrix. Due to their chemical similarity to starch, these were most likely to affect the glucose assay. The systematic error was computed as difference from zero.

The interference of **wood type** was evaluated by sampling 3 organs (1-year-old lignified twigs, 3-year-old branches, and lignified roots) of 4 species (*Acer pseudoplatanus* L.; *Cedrus deodara* G. Don; *Magnolia grandiflora* L.; *Pinus nigra* Arn.). These species were chosen because they display a diverse range of interfering factors, including different chemical composition [32], [33], [44], [45], reduced SC, variable ratio of parenchyma vs mechanical elements, and bark vs wood. Pooled wood powder of *Acer* twigs from the previous step (compared accuracy determination) was used as an internal reference (IR). SC was analysed concurrently in: i) sample, ii) sample + IR, and iii) IR. The systematic error was computed as the SC of the IR analysed together with the sample minus the SC of the IR alone. Measurements were carried out in triplicate.

The effect of **ethanol-solubles** removal was computed as the SC determined after extracting solubles minus the SC determined by analysing samples directly. Measurements were carried out in triplicate on the 12 wood types.

#### Precision and factors affecting precision

The precision for the 12 wood types (analysed in triplicate) and for the IR (analysed in triplicate for eight days) was computed as the coefficient of variation of the residual error after isolating the day effect (ANOVA, Genstat).

Three factors affecting precision were quantified: extraction of solubles (ES), day effect, and SC. The effect of **ES** was determined by analysing the 12 wood types in triplicate with and without ES and compared in an F-test. The **day effect** was isolated from the total variance of the IR analysed in triplicate for eight days (ANOVA, Genstat) and compared to the residual variance of the error in an F-test. The effect of **SC** was evaluated by regression analysis of S.D. against SC with use of statistical software (Genstat).

Glucose assay. The precision for the glucose assay was quantified by assaying glucose standards in triplicate for eight days. The day effect was isolated by ANOVA (Genstat) and compared to the residual error in an F-test.

## Results

### Accuracy

**Figure 2:**
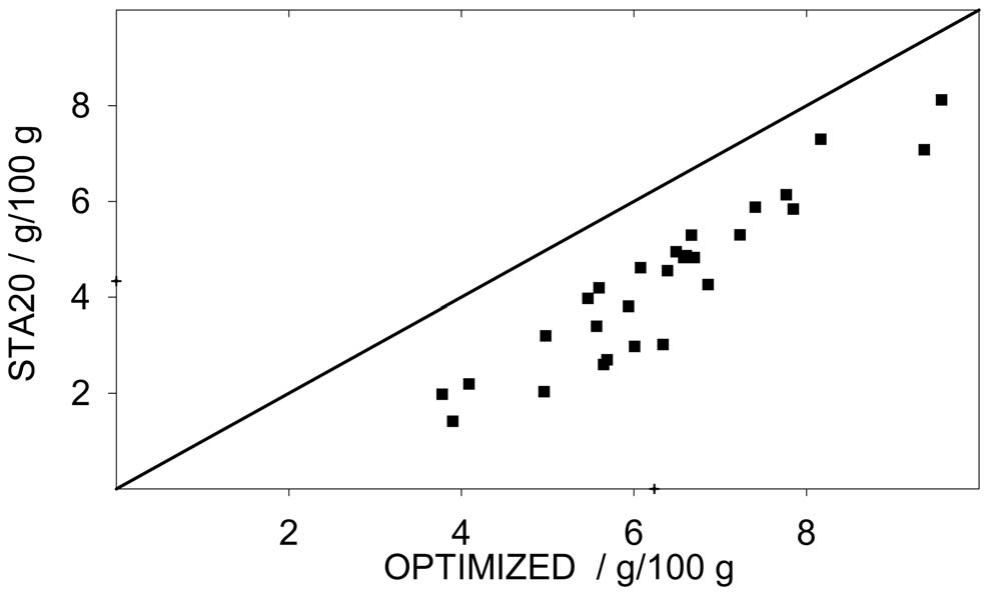
Compared SC of 28 samples of *Acer* twigs measured with two different methods: STA20 and the optimised protocol. Dots represent the average of five independent determinations (STA20) plotted against the average of four independent determinations (optimized) of the same sample. Crosses identify the grand mean for the two methods. The diagonal represents *y* = *x*.

Accuracy was tested by analysing samples at known SC (SKSC) and computed as systematic error (Table 2); relative to the mean it averaged 2.8%. Notably, the error was not significantly correlated with SC, i.e. the relatively high amounts of cellulose present in samples with low SC did not prevent starch detection, caused interference, showing that the optimised protocol has high specificity and sensitivity. Given these typical characteristics of enzymatic starch assay, there is circumstantial analogy between accuracy and digestion completeness. This observation allows the comparison of the accuracy of enzyme based methods directly by comparing the amount of starch detected in the same set of samples, a procedure which was also followed by [16]. This type of comparison is shown in Figure 2, where the optimised protocol is compared to another enzymatic analysis (STA20). Starch was analysed on 28 samples of *Acer* twigs. STA20 detected an average SC of 4.33 g/100 g, whereas the optimised protocol detected an average SC of 6.24 g/100 g (+ 44%) for the same set of samples, implying higher accuracy of the optimised protocol.

**Table 2:** Systematic error for starch analysis, measured on samples at known starch content (SKSC), prepared mixing standard grade corn starch and cellulose.

	Expected SC/g/100 g	Measured SC/g/100 g	Systematic error/g/100 g (%)
SKSC	4.688	4.819	0.131 (2.8)**
1.25 g/100 g	1.250	1.316	0.066 (5.3)
2.5 g/100 g	2.500	2.850	0.350 (14)**
5.0 g/100 g	5.000	5.172	0.172 (3.4)
10 g/100 g	10.000	9.937	0.063 (0.6)

Error was expressed as absolute SC value, and as relative to the expected SC (in brackets). Error was deemed significant for p<0.01 (**) in a single sample t-test. **SKSC** averages the 4 SKSCs. n = 7.

#### Factors affecting accuracy: Interferents

Interference was evaluated to test whether matrix polysaccharides or different types of wood would cause a systematic error in the starch determination. Interference was examined by adding matrix polysaccharides or different wood types to a reference starch analysis. In the case of matrix polysaccharides the reference starch determination was a sample blank, hence, the systematic error was computed as the difference from the expected value of zero. The interference of matrix polysaccharides was negligible (Table 3), in agreement with the observations on SKSCs described above. This result confirms the purity of the enzymes and agrees with a previous report where enzymes from the same vendor were used [27].

**Table 3:** Systematic error for the analysis of matrix polysaccharides and associated with the addition of 12 sample types to a reference starch determination.

	Systematic error/g/100 g
**Matrix**	
***Cellulose***	−0.015
***Pectin***	0.054
**Sample**	−0.029
***Organ***	
Twig	0.114
Mature	−0.073
Root	−0.129
Species*Organ	
Acer	−0.059
Twig	−0.269
Mature	0.099
Root	−0.008
Magnolia	0.214
Twig	0.403
Mature	0.451
Root	−0.212
Cedrus	0.150
Twig	0.472
Mature	0.087
Root	−0.110
Pinus	−0.423*
Twig	−0.150
Mature	−0.930
Root	−0.188

For matrix polysaccharides the error was not significant; n = 7. For different wood types the error was deemed significant in a t-test at p<0.05 (*) only when all *Pinus* organs were considered together; n = 3. **Sample** averages all samples. **Twig, Mature, and Root** average the 4 species. ***Acer, Magnolia, Cedrus, and Pinus*** averages the three organs.

To determine the interference from different types of wood, three different organs of four different species were added to a starch determination where *Acer* twigs were used as internal reference (IR). Interference was therefore computed as the difference in SC between the IR analysed alone and the IR analysed together with the interferent. The interference of **wood type** was generally low, and significant only when *Pinus* organs were considered altogether (Table 3). This shows that in spite of the wide range in SC (from 0.4 g/100 g in *Pinus* mature wood to 19.6 g/100 g in *Acer* roots, Table 4), and the diverse secondary metabolites [32], [33], [44] which may exert contrasting effects on the enzymes, the performance was only marginally affected. Furthermore, the sample chosen had a very wide range of wood/bark ratios, and parenchyma/lignified cells. These were higher in roots (which measured between 0.5 and 1.5 mm in diameter depending on the species) and in twigs (which measured between 3 and 8 mm in diameter depending on the species), while they were lower in mature wood (between 5 and 35 mm in diameter depending on the species).

**Table 4:** Precision for the starch analysis expressed as coefficient of variation (C.V.).

	Mean SC/g/100 g	C.V./%	V.R.
Sample	3.066	3.7	1.19
***Organ***			
Twig	1.610	2.5	0.15^††^
Mature	1.659	8.1	1.69
Root	5.930	2.3	1.73
Species*Organ			
Acer	9.834	1.7	2.78^*^
Twig	4.879	0.8	0.20
Mature	4.997	2.3	1.81
Root	19.63	1.1	6.31^**^
Magnolia	0.839	5.9	0.23^†^
Twig	1.004	5.0	0.36
Mature	0.799	5.8	0.31
Root	0.715	2.2	0.03^†^
Cedrus	0.157	83	1.67
Twig	−0.009	-	0.04^†^
Mature	0.437	41	4.51^*^
Root	0.042	133	0.45
Pinus	1.435	2.2	0.09^††^
Twig	0.565	2.1	0.04^†^
Mature	0.402	7.9	0.29
Root	3.338	0.9	0.23

To compare precision, the variance for the sample was divided by the variance for the internal reference. In an F-test the variance ratio (V.R.) was significantly higher than unity (lower precision for sample) at p<0.05 (*), p<0.01 (**) or significantly lower than unity (higher precision for sample) at p>0.95 (†), p>0.99 (††). **Sample** averages all samples. **Twig, Mature and Root** average the 4 species. ***Acer, Magnolia, Cedrus and Pinus*** average the three organs. n = 3.

Another important factor affecting accuracy was extraction of solubles (**ES**), which increased SC in all cases tested (Table 5), In absolute terms, the increase in SC (average effect in Table 5) ranged from 0.05 g/100 g in *Magnolia* twigs to 1.11 g/100 g in *Acer* roots. However, the relative effect was lower in samples with higher starch content. In fact, the relative effect ranged from 5% in *Magnolia* twigs to 1480% in *Cedrus* roots. Interestingly, in *Cedrus* twigs without ES, no starch was detected. In light of these observations and the unlikeliness of matrix interference highlighted above, we infer that ES increased accuracy and we propose it as a powerful tool to manipulate the performance of the analysis (see Discussion).

**Table 5:** Effect of extraction of solubles (ES) on analytic performance.

	SC/g/100 g	Average effect/g/100 g (%)	C.V./%	V.R.
Sample	3.639	0.572 (19)**	2.4	0.61
***Organ***				
Twig	2.176	0.566 (35)**	3.5	3.55^*^
Mature	1.991	0.332 (20)**	3.1	0.21
Root	6.748	0.818 (14)**	1.7	0.66
Species*Organ				
Acer	10.668	0.834 (8)**	1.0	0.38
Twig	5.928	1.049 (22)**	0.2	0.07
Mature	5.335	0.338 (7)*	0.8	0.14
Root	20.74	1.116 (6)*	0.7	0.46
Magnolia	1.060	0.221 (26)**	8.4	3.18
Twig	1.058	0.054 (5)	5.3	1.25
Mature	0.947	0.148 (19)	6.2	1.57
Root	1.175	0.460 (64)*	8.1	38.0^*^
Cedrus	0.907	0.751 (478)**	11.0	0.56
Twig	1.022	1.031 (−)**	10.3	36.2^*^
Mature	1.037	0.600 (137)*	4.5	0.07
Root	0.663	0.621 (1479)*	12	2.08
Pinus	1.919	0.484 (34)**	2.3	2.02
Twig	0.697	0.132 (23)	5.0	4.49
Mature	0.647	0.245 (61)**	7.9	1.28
Root	4.412	1.074 (32)**	0.3	0.93

The effect on starch detection was calculated by subtracting the SC determined without pretreatment (Table 4) from the starch content (SC) determined after ES. In brackets the effect is expressed as per cent increase in SC. The effect was deemed significant for p<0.05 (*), p<0.01 (**). The precision for samples analysed after ES was expressed as coefficient of variation. The precision after ES was compared to the precision for the same samples (Table 4) analysed without pretreatment (expressed as variance ratio, V.R.). In an F-test, V.R. was significantly higher than unity (ES decreased precision) at p<0.05 (*). **Sample** averages all samples. **Twig, Mature, and Root** average the 4 species. ***Acer, Magnolia, Cedrus, and Pinus*** average the three organs; n = 3.

## Precision

### 

To study whether precision was affected by the type of sample, we analysed the SC of different wood types and computed the dispersion of the results as the coefficient of variation (C.V.). The C.V. ranged between 0.8% in *Acer* twigs to 133% in *Cedrus* roots, but this high relative value was due to the low SC in *Cedrus*. The bulked C.V. for all woody samples tested (samples + IR, Tables 4 and 6) averaged a remarkable 3%.

**Table 6:** Precision (expressed as C.V.) and day effect (expressed as variance ratio, V.R.) for starch content of the internal reference and absorbance of the glucose standards.

	Mean	C.V./%	Variance (S^2^)	V.R.
Internal Reference (SC)	4.852	2.1	0.0106	-
Day	-	-	0.1060	10.0^**^
Glucose standard (Absorbance)	0.475	0.8	1.43·10^−6^	-
Day	-	-	1.07·10^−3^	75**

In an F-test the V.R. was deemed significant at p<0.01 (**). n = 3, days  = 8.

To compare the precision for different samples we calculated the ratio between the sample and internal reference variance of the error (variance ratio, V.R., also referred as F-ratio). V.R. ranged from 6.31 for *Acer* roots (i.e. the analysis was less precise for the sample than for the IR) to 0.04 for *Pinus* twigs (i.e. the analysis was more precise for the sample than for the IR). This wide range of V.R. can be mainly attributed to the small sample size (n = 3, but see also below the effect of SC). In fact when all samples were bulked, V.R. was close to 1 (V.R. = 1.19, Table 4).

#### Factors affecting precision

Different factors may affect the precision of the analysis; that is, they may cause higher or lower dispersion of the results. Here, three factors affecting precision were evaluated: extraction of solubles (ES), day effect, and SC.

The precision of the starch analysis after ES was calculated as coefficient of variation, which was, on average, 2.4% (Table 5), not significantly different from the average C.V. of all samples without ES (3.5%). The effect of ES on single samples was evaluated by calculating the ratio between the variance of the error of the direct analysis and the variance of the error of the analysis after ES (V.R., Table 5). No significant difference was found in 10 samples out of 12, but in three cases (*Magnolia* root, *Cedrus* twigs, and when all twigs were averaged), V.R. was significant. This means that the possibility that ES decreases the precision of the analysis should be taken into account when designing the experiment (see Discussion).

A critical component of the error is the ‘day effect’, which is the fraction of the total variance attributable to daily fluctuations. The ‘day effect’ can be isolated if the experiment is purposely designed, and may be significant or not, depending on the type of analysis. Table 6 shows the isolation of the day effect from the total variance of the error for the IR analysed in 8 different days. The day effect (i.e. the between-day variance) was divided by the residual variance of the error (V.R.) and proved significant in an F-Test. A similar result was obtained for the 28 *Acer* twigs (not shown). The day effect cannot be eliminated by using a standard, because an accepted, *intact* wood sample at *known* starch content is not available. However, we propose an alternative statistical approach (see Supporting Information).

**Figure 3:**
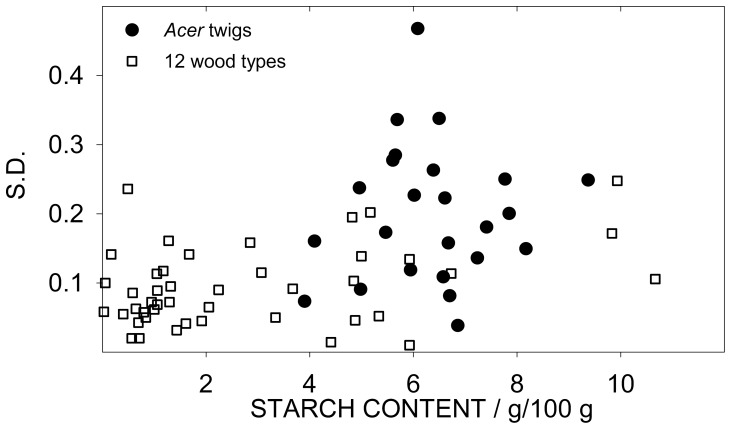
Relationship between standard deviation and starch content (SC). Dots represent the 28 *Acer* twigs (n = 4); squares represent the 12 wood types (n = 3).

Another effect which may affect precision is the starch content of the sample. That is, samples with lower SC have lower S.D. (e.g. shown as low V.R. of *Pinus*, Table 4). To study the effect of the SC on precision, we tested the correlation between SC and S.D. of the error. As shown in Figure 3 there is no significant correlation between **SC** and S.D. in the 28 *Acer* samples and in the 12 wood types when taken altogether. However, when each wood type was individually tested, a significant positive correlation between SC and S.D. was found. This correlation has important implications for data analysis which are detailed in the Discussion.

## Glucose assay

**Figure 4:**
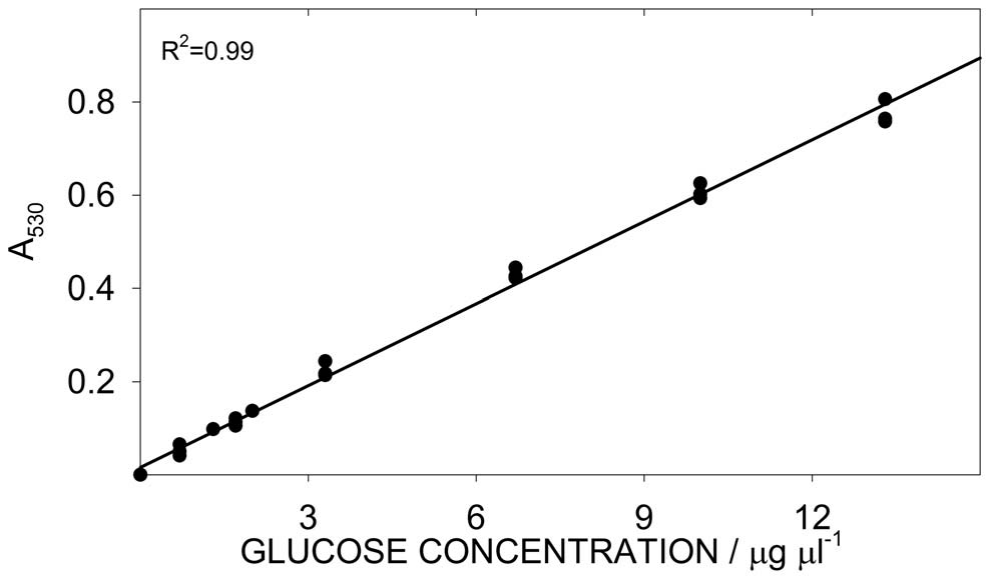
Calibration curve for glucose determination.

The glucose assay response was linear between (0 and 40) µg of glucose (Figure 4), corresponding to a concentration of (0 to 13.3) µg ml^−1^ in the cuvette and to a concentration of (0 to 67) µg ml^−1^ in the volume occupied by sample + water (0.6 ml, see Table 1). Similarly to the overall starch determination, we evaluated the precision of the glucose analysis by analysing the glucose standard in triplicate for 8 days (Table 6). The C.V. for the glucose assay averaged 0.8%, while the **day effect** was highly significant. To compensate for this effect, fresh glucose standards were prepared every day (as described in Box 2), instead of using a single calibration curve (Figure 4).

## Detection limits

Starch detection may be constrained by i) the capacity of the enzymes to hydrolyse starch and by ii) the capacity of the glucose assay to detect glucose. As we have described above, pure starch was easily hydrolysed, so the hydrolysing capacity (i) did not limit the analysis at high SC. Conversely, at low SC the hydrolysing capacity was affected by interferents, which have been extensively described. In the glucose assay (ii), it is of critical importance to maintain the colorimetric response within the linearity limits described above. Conservatively, the bottom and top 10% of these limits (Figure 4) can be discarded. With the suggested dilutions (Box 2), an initial starch mass between ≈0.6 and ≈6 mg (corresponding to a SC between ≈1.5 and 15 g/100 g in a sample mass of 40 mg) would yield a colorimetric response within the conservative limits of linearity. Of course, there are several expedients to maintain the colorimetric response within the linearity limits. For instance, the sample mass can be reduced or increased. Although there are no issues associated with this strategy, provided that a suitable balance is used to weigh the samples, we preferred to keep the sample mass in the same order of magnitude to that indicated by the standard protocol (we suggest 40 mg, the standard protocol indicates 100 mg [34]). Instead, we adjusted the dilution of the sample in the glucose assay, which can be varied between 0 and 600 µl (suggested 60 µl). An additional regulation point is the final digestion volume (suggested 10 ml, minimum of 3.15 ml when no water is added). For instance, when pure starch was analysed, the volume of digestate added to the glucose assay was reduced to 5 µl (we used an appropriate syringe, and 595 µl of water were also added to reach the final volume of 600 µl). We preferred these dilutive adjustments as they do not influence the digestion conditions and offer wider adjustment opportunities. In an extreme case, if the digestion mixture is not diluted and 600 µl of digestate are added to the glucose determination, an initial starch mass of just 21 µg would yield a colorimetric response within the conservative linearity limits.

## Speed

One operator with a single set of equipment could assay 35 samples in 8 h.

## Discussion

The digestion conditions (i) and the glucose assay (ii) of a standard method for the analysis of starch used in the food and feed industry [18], [31], [34] were optimised for the analysis of starch in wood samples. In the (i) optimised digestion, the amount of amyloglucosidase added in the second step was increased from 0.1 to 0.15 ml, the digestion time was increased, and a magnetic bar, which kept the slush in suspension throughout, was added. These modifications increased the duration of the digestion by ≈25 min and increased the starch detection in wood samples, compared to the unmodified standard protocol by ≈40%. The addition of the magnetic bar alone determined a ≈30% increase in starch detection. This expedient was suggested by [14], but it is not part of the standard protocols as it is not necessary for the complete digestion of food and feed material [18], [34]. For the glucose assay (ii) we followed a protocol that had already been optimised for the analysis of wood [26], with some modifications. The protocol of [26], instead of using aminoantipyrine as a dye, as suggested by Megazyme [34], used o-dianisidine (see also [16]), and introduced the addition of sulphuric acid at the end of the reaction to stabilise the colour and shift the spectra away from the interference of plant pigments. These modifications have recently been included in the Sigma protocol [41]. Here, we proposed a different dilution order, which is intended to synchronize the reaction time of all samples. We also increased the reaction time from 30 to 45 min, as we found that this made the colour development more uniform between batches and therefore reduced the noise.

The resulting optimised protocol was then characterized. To the best of our knowledge this is the first report in which the analytical performance of a similar protocol is evaluated in terms of precision and accuracy for wood samples. We acknowledge that the performance of the analysis will ultimately differ in each lab implementing the optimized protocol. However, knowing the reasonably attainable performance, and the possible caveats, may be useful to help choose one protocol over others. To further facilitate this task we used ordinary equipment, available in any lab. Furthermore, the experience acquired in this study can be highly valuable to orient experimental setup and data treatment.

### Wood starch reactivity: Need for a dedicated protocol

Wood starch demonstrated reduced digestibility compared to food starch. Corn starch in SKSC was promptly digested in all conditions tested (e.g. ≈94% detection with STA20 and ≈100% with the optimised protocol, Table 2). On the other hand, when wood samples were digested, different methods resulted in contrasting digestion completeness (e.g. STA20, as compared to the optimised method, resulted in 30% lower detection for *Acer* twigs, Figure 2). This contrasting reactivity between different starch granules is consistent with results of previous studies. For instance, with an enzymatic starch analysis 99.4% of potato starch was detected already after 24 hours of hydrolysis, but when the reaction time was doubled, detection in wood samples increased in different replicates by 5% to 28% respectively [26]. Another study found different reactivity of pea starch as compared to maize or potato starch [25].

The contrasting digestibility of starch may reflect the physiological role of starch in wood as compared to the starch from storage organs and leaves. For instance, starch in leaves is generally mobilized overnight while wood starch represents a long term energy reserve, mobilized on a seasonal cycle [6]. From these observations, we deduced that the digestion was the most delicate part of the analysis and the capacity of the enzymes to reach starch granules, and to *quantitatively* convert them into glucose was the key determinant of analytic performance. An optimized digestion, which is capable of mobilizing starch granules locked within the complex wood matrix is therefore critical for the analysis of starch in wood.

### Implications for experimental setup and data treatment

The lower reactivity of wood starch implies that the accepted standard for food and feed starch analysis (pure corn starch) cannot be used as a standard for wood starch analysis. Since the two types of starch are not equally digested, the systematic error of the analysis cannot be assumed equal for standard starch and wood samples and therefore the high accuracy measured on SKSC is not sufficient to prove high accuracy for wood samples. For this reason accuracy for wood samples had to be evaluated in relative terms by comparing the SC detected with two methods (Figure 2, [16]).

Another disadvantage of the lower reactivity of wood concerns the treatment of the day effect. Conventionally, the day effect is eliminated by including in each analysis a standard, and by scaling the measured quantity to the values found for the standard. For example, in the glucose assay, glucose standards were included in triplicate (Box 2), and the glucose content of samples was calculated by scaling the absorbance observed for the samples to the absorbance observed for the standards. In this way the result of the analysis was corrected for any systematic error that may have resulted in a daily fluctuation in absorbance. Since an *intact* wood sample at *known* starch content is not available, this conventional procedure was not implementable for the starch analysis. Instead, the day effect was isolated statistically after the experiment was purposely designed. The statistical procedure and the experimental design for the isolation of the day effect are detailed in Supporting Information.

### On whether to extract solubles (ES)

We showed that removal of ethanol-solubles improved starch detection. This further highlights the differences between wood and food and feed, where ethanol-solubles are mainly monosaccharides and dextrins, and their removal results in lower starch detection [18]. We propose ES as a tool to increase accuracy, although, the magnitude of the increase may depend on the species and on the SC itself (Table 5). The appropriateness of ES should be carefully evaluated because: i) ES takes 1 h; ii) ES adds to the complexity of the assay; and, as a result, iii) ES may result in lower precision (Table 5).

The appropriateness of ES will therefore depend on the purpose of the analysis and on the sample type. a) When precision is important, as in hypothesis-testing (e.g. treatment vs. control, where the statistical power of the test correlates to precision), or when evidence suggests that the accuracy gain would be minimal (e.g. as in *Acer* roots), it may be appropriate to analyse samples directly and thereby avoid the risk of amplifying error. b) When accuracy is decisive (e.g. when the SC is used to derive other physiological information, inter-lab studies, or time courses), or when evidence suggests that the accuracy gain would be significant (e.g. as in *Cedrus* twigs), ES may be appropriate. c) When the analysis is also intended to evaluate the soluble sugar content, ES is indispensable. Sugar analysis, in fact, is performed on the ethanol extract. Describing the techniques for sugar analysis goes beyond the scope of this paper; we mention only that the complexity of the sugar analysis may vary greatly, from a simple colorimetric determination of the total sugar content (e.g. refs in Box 1), to an enzymatic determination of single or multiple sugars e.g. [46], to a complete HPLC quantification of all sugars extracted e.g. [21], [47].

If ES is to be included before the starch assay, the risk of precision loss resulting from ES can be minimised with a higher number of replicates.

### Suitability of the optimised protocol

- The optimised protocol was suitable for the determination of SC in food and feed. The accepted standard for calibrating food and feed starch analysis is standard starch. The optimised protocol performed well on standard starch (Table 2), generally exceeding the performance of the other two methods used (standard and STA20).
- The optimised protocol was suitable for the determination of SC in wood. The optimised protocol performed better than the standard method and STA20 (Figure 2) on wood samples. The optimised protocol showed similar accuracy and precision for various lignified organs of different species (with different amounts of secondary metabolites; living vs. dead cells; parenchyma vs. mechanical elements, etc.).
- The optimised protocol can conveniently be used as the sole analytical protocol when the SC of different organs (lignified or not) is being measured, to facilitate comparison between results. In fact, the processing time was similar to the standard protocol (c. 40 min higher on a daily basis).
- The optimised protocol was highly flexible and could detect between 40 mg and 21 µg of starch. With the proposed dilution it could detect from (0.6 to 6) mg of starch in the sample. For instance, these limits would include the ≈1.8 mg of starch expected in ≈6 mg of dry pea powder extracted non-destructively from single pea seeds with a micro-analytical method [25]. When using different dilutions the limits of detectability can be greatly extended: the maximum starch concentration was 100% (pure starch) while the potential minimum quantity of starch detectable was ≈21 µg. Note that a balance with an appropriate sensitivity should be used when weighing small amounts of sample.

## Conclusions

A standard protocol for starch analysis in food and feed was optimised to improve precision and accuracy for the analysis of wood. The optimisation included key modifications to starch digestion, and glucose assay together with an appropriate experimental design and data treatment. The performance of the optimised protocol was tested with 430 starch analyses. The optimised protocol proved to be remarkably precise and accurate (3%), suitable for a high throughput routine application (35 samples per day). The optimised protocol can be used for the determination of starch in the most diverse plant material including lignified organs (roots, shoots, mature wood) of coniferous and flowering plants and non-lignified organs, such as leaves and fruits. Solubles can be extracted prior to starch analysis, either for the determination of sugar content or as a tool to increase accuracy, but an additional number of replicates may be required. The upper and lower starch detection limits were 40 mg and 21 µg. A step-by-step example of experimental design and data analysis is reported in Supporting Information.

## Supporting Information

Supporting Information S1 **Experimental design and statistical separation of the day effect.**
(DOCX)

Supporting Information S2 **Example of starch analysis design.**
(DOCX)

## Abbreviations

C.V.: coefficient of variation
ES: extraction of solubles
GS: glucose standard
IR: internal reference
SAB: sample blank
S: sample
SC: starch content
S.D.: standard deviation
SKSC: sample at known starch content
STB: standard blank
