# Supplementary material for: Evaluation of a High Throughput Starch Analysis Optimised for Wood
Source: PLoS One. 2014 Feb 11;9(2):e86645. doi: 10.1371/journal.pone.0086645 (PMC3921133; doi:10.1371/journal.pone.0086645)
Supplement: Supporting Information S2 — Example of starch analysis design. (DOCX) [file pone.0086645.s002.docx]

Supporting information S2: Example of starch analysis design

*The aim is to gain statistical evidence for the difference in SC between two broadleaf woody samples, S1 and S2.*

In a preliminary experiment SC for sample S1 was 3.7 g / 100 g and SC for sample S2 was 3.85 g / 100g. The difference between means will be significant if [[1](#_ENREF_1)]

| $\text{n>2}\left(\frac{\text{t}\times\sigma}{\left\vert\overline{x}_{(S1)}-\overline{x}_{(S2)}\right\vert}\right)^{2}$ | Equation 2 |
| --- | --- |

Where t has the degrees of freedom of the error at the desired probability (here 0.05), 𝜎 is the population standard deviation and the denominator represents the difference between sample means. We can get an estimate of 𝜎 by multiplying by the expected mean (3.7 g / 100 g) times the sample C.V. (3.7 %) (in Table 3), resulting in 𝜎 = 0.13 g / 100g. Taking a conservative t value of 2.5 and a difference between means of 0.15 g / 100g (3.7 – 3.85) g / 100g, n would need to be >10. For the sake of example, we decide to split the experiment over two days. n is increased from 10 to 12 to compensate for the degree of freedom lost during day-blocking. Each day, each sample is therefore analysed six times.

The aim is to gain statistical evidence for the separation of the two means, so precision has the priority over accuracy. Considering that samples are taken from broadleaf species with relatively high SC and low expected soluble interference, we decide to analyse samples directly without extracting ethanol-solubles. Table S1-1 shows the dilution for the glucose determination. The results of the analysis, expressed as absorbance and starch concentration (calculated with Equation 1) are also reported in Table S1-1. The additional error generated by splitting the experiment over two days (day effect) can be eliminated because day-blocks were homogeneous. Since the SC of the two samples is very similar, the additive separation of error performed by an ANOVA is appropriate. Data are arranged as in Table S1-2 and subject to ANOVA (SC is the variate, sample is the treatment factor, and day is the block factor). The ANOVA table (Table S1-3) shows the isolation of a significant day effect and a significant difference between samples, as was the aim. Without the isolation of the day effect the difference between samples would not be significant.

In this example, the ANOVA studied the relationship between the variability generated during the analysis and the difference between the mean SC for the two samples S1 and S2. No information on the population from which samples were collected is available. Therefore, the conclusion about the significance cannot be extended to the groups from which the two samples were collected. To gain evidence for the difference between two groups, a different experimental design is required (refer to a text of experimental design, e.g. [[1](#_ENREF_1)]).

**References:**

1. Clewer AG, Scarisbrick DH (2001) Practical statistics and experimental design for plant and crop science. Chichester, New York: J. Wiley.

**Table S2-1:** Dilution table, absorbance and corresponding SC for samples S1 and S2

|  |  |  |  |  |  |  | **Day 1** | | **Day 2** | |
| --- | --- | --- | --- | --- | --- | --- | --- | --- | --- | --- |
|  | **Water /  μl** | **Supernatant /  μl** | **Reagent 3 /  μl** | **Standard /  μl** | **H_2_SO_4_ 75 % /  μl** | **Total Volume / μl** | **A** | **SC /  μg/mg** | **A** | **SC /  μg/mg** |
| **S1-1** | 540 | 60 | 2000 | - | 400 | 3000 | 0.233 | 34.48 | 0.237 | 35.44 |
| **S1-2** | 540 | 60 | 2000 | - | 400 | 3000 | 0.235 | 35.02 | 0.236 | 35.29 |
| **S1-3** | 540 | 60 | 2000 | - | 400 | 3000 | 0.248 | 38.22 | 0.248 | 38.33 |
| **S1-4** | 540 | 60 | 2000 | - | 400 | 3000 | 0.242 | 36.72 | 0.244 | 37.20 |
| **S1-5** | 540 | 60 | 2000 | - | 400 | 3000 | 0.237 | 35.49 | 0.247 | 38.05 |
| **S1-6** | 540 | 60 | 2000 | - | 400 | 3000 | 0.237 | 35.44 | 0.241 | 36.40 |
| **S2-1** | 540 | 60 | 2000 | - | 400 | 3000 | 0.253 | 39.32 | 0.261 | 41.46 |
| **S2-2** | 540 | 60 | 2000 | - | 400 | 3000 | 0.260 | 41.16 | 0.269 | 43.29 |
| **S2-3** | 540 | 60 | 2000 | - | 400 | 3000 | 0.257 | 40.46 | 0.262 | 41.57 |
| **S2-4** | 540 | 60 | 2000 | - | 400 | 3000 | 0.262 | 41.71 | 0.269 | 43.29 |
| **S2-5** | 540 | 60 | 2000 | - | 400 | 3000 | 0.260 | 41.25 | 0.263 | 41.90 |
| **S2-6** | 540 | 60 | 2000 | - | 400 | 3000 | 0.248 | 38.28 | 0.255 | 39.95 |
| **Sample Blank** | 540 | 60 | 2000 | - | 400 | 3000 | 0.092 | - | 0.092 | - |
| **Glucose Std 1** | 400 | - | 2000 | 20 | 400 | 3000 | 0.549 | - | 0.552 | - |
| **Glucose Std 2** | 400 | - | 2000 | 20 | 400 | 3000 | 0.559 | - | 0.562 | - |
| **Glucose Std 3** | 400 | - | 2000 | 20 | 400 | 3000 | 0.569 | - | 0.572 | - |
| **Standard Blank** | 600 | - | 2000 | - | 400 | 3000 | 0.069 | - | 0.072 | - |
|  |  |  |  |  |  |  |  |  |  |  |

**Table S1-2**: Data organised for the statistical treatment

| **Sample** | **Day** | **Starch** |
| --- | --- | --- |
| S1 | 1 | 3.448 |
| S1 | 1 | 3.502 |
| S1 | 1 | 3.822 |
| S1 | 1 | 3.672 |
| S1 | 1 | 3.549 |
| S1 | 1 | 3.544 |
| S1 | 2 | 3.932 |
| S1 | 2 | 4.116 |
| S1 | 2 | 4.046 |
| S1 | 2 | 4.171 |
| S1 | 2 | 4.125 |
| S1 | 2 | 3.828 |
| S2 | 1 | 3.544 |
| S2 | 1 | 3.529 |
| S2 | 1 | 3.833 |
| S2 | 1 | 3.720 |
| S2 | 1 | 3.805 |
| S2 | 1 | 3.640 |
| S2 | 2 | 4.146 |
| S2 | 2 | 4.329 |
| S2 | 2 | 4.157 |
| S2 | 2 | 4.329 |
| S2 | 2 | 4.190 |
| S2 | 2 | 3.995 |

**Table S1-3**: ANOVA table for the data of the example

|  |  |  |  |  |  |
| --- | --- | --- | --- | --- | --- |
| Variate: Starch |  |  |  |  |  |
|  |  |  |  |  |  |
| Source of variation | d.f. | s.s. | m.s. | v.r. | F pr. |
|  |  |  |  |  |  |
| day stratum | 1 | 1.38037 | 1.38037 | 83.20 |  |
|  |  |  |  |  |  |
| day.*Units* stratum |  |  |  |  |  |
| Sample | 1 | 0.08872 | 0.08872 | 5.35 | 0.031 |
| Residual | 21 | 0.34843 | 0.01659 |  |  |
|  |  |  |  |  |  |
| Total | 23 | 1.81751 |  |  |  |
|  |  |  |  |  |  |
